# Editorial for the Special Issue: Feature Reviews in Adipokines

**DOI:** 10.3390/biomedicines13081916

**Published:** 2025-08-06

**Authors:** Christa Buechler

**Affiliations:** Department of Internal Medicine I, University Hospital Regensburg, 93053 Regensburg, Germany; christa.buechler@klinik.uni-regensburg.de

Adipokines are a growing group of bioactive peptides and proteins that play a significant role in metabolism. As adipokines are predominantly expressed in adipose tissue, they are most extensively studied in relation to obesity and obesity-associated metabolic diseases [1,2]. Obesity is a state of chronic, low-grade inflammation, and adipokines play a role in regulating this inflammatory state [3,4]. Dysfunctional adipose tissue in obese individuals is associated with an altered adipokine profile, which contributes to inflammation, metabolic diseases, and certain types of cancers [3]. Insulin resistance is a characteristic of metabolic diseases, but it also occurs in patients with severe inflammation [5] and in cancer patients [6,7]. Adipokines regulate glucose-induced insulin secretion, which is reduced by leptin and improved by adiponectin [8]. Adipokines such as adipsin, apelin, chemerin, fibroblast growth factor 21, lipocalin-2, resistin, and visfatin also affect beta-cell function and insulin secretion [8]. Omentin-1 increases the proliferation of islet beta-cells [9] and also improves insulin resistance in peripheral tissues [10]. Therefore, omentin-1 could be used to treat glucose intolerance. Current studies indicate that omentin-1 is a potential biomarker for metabolic syndrome, obesity, diabetes, atherosclerosis, ischaemic heart disease, and inflammatory diseases [10].

Omentin-1 levels were found to be reduced in gestational diabetes mellitus, which is characterized by high blood sugar levels during pregnancy due to insulin resistance [11]. Pregnancy is associated with metabolic changes, and the levels of various adipokines fluctuate during a normal pregnancy. Several studies have been published that measured adipokines in maternal and/or cord blood and showed associations with pregnancy outcomes. Kabbani et al. summarized these studies, concluding that adipokines may play a significant role in the complex physiological changes that occur during pregnancy, with implications for managing pregnancy-related health conditions [11].

Regular exercise improves insulin response [12], and it should also be noted that exercise affects adipokine levels, systemic inflammation, and oxidative stress, thereby improving health. However, the impact of different diets on insulin activity is not well understood [13], and the effect of exercise on systemic adipokines is unclear [3].

Adipokines such as fibroblast growth factor 21 and leptin have been studied for their potential therapeutic benefits in humans. Treatment with fibroblast growth factor 21 agonists has shown promise in metabolic diseases such as obesity, due to its beneficial effects on insulin response, lipid levels, and/or food intake [14]. The patients suffering from congenital leptin deficiency and obesity experienced a reduction in their body weight as a result of leptin [14].

Further clinical evidence supports the use of adipokines as a treatment and/or a biomarker for various diseases [14]. Adipokines can be detected not only in the blood, but also in urine and feces. Current experimental findings regarding the analysis of fecal and urinary adipokine concentrations suggest their potential as disease biomarkers [15]. For example, elevated levels of adiponectin, leptin, lipocalin-2, and interleukin-6 have been observed in urinary samples from patients with renal disorders. Additionally, a positive correlation has been found between elevated urinary chemerin levels and higher fecal and urinary lipocalin-2 levels, with fecal calprotectin levels acting as a marker of disease activity in patients with inflammatory bowel diseases [15]. Furthermore, urinary interleukin-6 levels may serve as biomarkers for kidney transplant rejection, while elevated fecal interleukin-6 levels are indicative of decompensated liver cirrhosis [15].

Adipokines have multiple effects and appear to play a role in various diseases [3]. Rheumatoid arthritis affects millions of people, and hundreds of studies have analyzed the role of adipokines in patients with this condition [16]. Rheumatoid arthritis is a chronic inflammatory disease characterized by joint involvement and extra-articular manifestations. There is now convincing evidence that adipokines contribute to the pathogenesis of rheumatoid arthritis. Furthermore, adipokines such as leptin, adiponectin, and visfatin in blood and/or synovial fluid may serve as biomarkers for disease activity, therapeutic response, and prognosis [16]. Identifying adipokines that could be effective therapeutic targets could provide new opportunities for treating rheumatoid arthritis [16]. Chronic rheumatoid arthritis can result in bone loss and osteoporosis [17]. Adipokines such as omentin-1 enhance osteoblast differentiation and inhibit osteoclast differentiation. In contrast, leptin and chemerin inhibit osteoblast formation, demonstrating the potential of adipokines as targets for osteoporosis therapy [18]. Blood adipokine levels have also been analyzed as potential osteoporosis biomarkers, but so far these findings are not conclusive [18].

There is convincing evidence that obesity contributes to more severe disease outcomes in individuals infected with severe acute respiratory syndrome coronavirus 2 (SARS-CoV-2). Accordingly, adipokines have been measured in the blood of patients with SARS-CoV-2 infections [19]. Although a growing number of studies have examined adipokine levels in patients with SARS-CoV-2 infections, a significant limitation of the existing data is the lack of comparisons between patients, with similar illness severity, that have SARS-CoV-2 infections and those that do not have the infection. Consequently, it is difficult to identify changes that are specifically associated with the severity and outcomes of COVID-19 [19].

Finally, adipokines are being studied in animals because obesity is also a health concern in domestic cats [20]. Marshall, Chen, and Viloria-Petit’s review article summarized studies on adipokines in feline mammary cancer [20]. The authors concluded that the similarity in the pathophysiology of obesity in humans and felines makes domestic cats a suitable model for investigating the effects of obesity on breast cancer development [20].

Overall, the review articles included in this Special Issue demonstrate the variety of adipokines and their numerous functions in health and disease. Advances in this field could lead to better patient management in terms of diagnosis, prognosis, and the development of new therapies.
